# Selections

**Published:** 1897-12

**Authors:** 


### SELECTIONS.

RAYMOND, in the *Horse World* of November 19th, writes: "It is reported that the *American Veterinary Review* recently referred to the 'trotter' as a horse in which pedigree counts all, conformation little, and beauty nothing, and whose injudicious and limitless breeding has nearly ruined the horse-industry of the country . . ."

It is to be feared that the *American Veterinary Review* is in need of a man, in an editorial capacity, who has sense enough to look around and see for himself before rushing into print with such asinine assertions, utterly lacking in truth. Such beautiful trotters as Daredevil, 2.09¾; Pilot Boy, 2.09¼; Elloree, 2.09¼; Valence, 2.12¼; The Abbott, 2.11¼; Grace Hastings, 2.08; Arion, 2.07¾, and many others that might be mentioned, give the lie to the assertion regarding the lack of beauty, while the severe racing campaigns made by these horses tell of a conformation serviceable in the extreme. The many sales being made daily of trotting-bred carriage and coach pairs, many for export, show the utter falsity of any assertion reflecting on the usefulness of the American trotter for other than racing purposes. Recently, at Kansas City, R. M. C. Lord paid $4000 for the trotting-bred Brilliance and Botanic. Brilliance was sired by Reno's Baby, 2.25½, dam by Pilot Mambrino, and Botanic was sired by Counselor, 2.12½, dam by Charles Caffrey. When the editor of the *American Veterinary Review* can point out some team sired by a hackney or a French coach sire that has sold for a larger price, then will its assertions regarding the worthlessness of trotting-blood carry some weight. The largest winner in the harness classes at the recent Chicago Horse-show was Dr. George S. Gagnon, of New York, and in speaking of the breeding of horses he showed he said: " I have not an animal in my stable but is trotting-bred. Northlight, for instance, which, with his mate, Great Cæsar, won the C. P. Kimball special to victoria, was campaigned, and has a trotting record of 2.17¼. He is sired by Twilight, son of Hambletonian 10, and his dam is Sally Western, by Empire, son of Mambrino Patchen. Here is another, McKusick, who won the blue in class 232, for horse and trap suitable for lady for park use. He is trotting-bred, has been trained, and has a record. Without any exception, every horse that I have shown is bred in trotting-lines, and most of them are standard and registered. H. M. Tichenor & Co., of Chicago, were the second largest winners, and all their horses were trotting-bred. The pair of chestnuts, Challenger and Chancellor, are bred in the stoutest lines. These two, with Larkspur and Howard, won the prize for four-in-hand to coach, and Mr. Tichenor was offered $7500 for the four by a New York man, but declined to consider the proposition. Other ribbons fell to their lot during the show, and in the end Challenger won the $1000 prize for championship of the harness classes, mare or gelding, any age or height. This handsome gelding, the champion in the ring of the greatest horse-show ever held, was bred at Streator, Ill., and was sired by Oneida, son of Nutwood, 2.18¾, dam Babe, by Hambletonian 10. He won first prize as a high-stepper at Kansas City, and first prize in the lead of park tandems at the same show. With Chancellor, he won first for pair of high-steppers. Chancellor, that won first for best park mare or gelding, is a trotter, though not as stoutly or fashionably bred as his mate. He was raised at Wamago, Wis., and was sired by George Chief, a son of Milwaukee, dam by David Hill, Jr. He was trained and took a record of 2.37½ under the name of Red Chief. Larkspur, one of the prize four-in-hand, and that also won first in the class for town mare or gelding, comes from southern Ohio, and Mr. Tichenor says is trotting-bred; while Harvard, the other leader, was raised at Rockford, Ill., and was sired by Star Hambletonian. Wae Bell and Ben Bow, winners in the class for pair of park horses, are exceptionally well-bred ones. The former was sired by the dead Bell Boy, 2.19¼, son of Electioneer, and the latter by Aladdin Wilkes. Sir Walter, another of the Tichenor prize-winners, was sired by Swigert, and has trotted a mile in 2.31. If beauty and conformation do not count in the show ring, where will one go to find where those qualities do? And, if, conceding that beauty and conformation do not count in the show-ring, how much truth is there in the assertions made by the *American Veterinary Review?*

